# Development and Validation of a Stability-Indicating HPLC Method for the Simultaneous Determination of Sulfadiazine Sodium and Trimethoprim in Injectable Solution Formulation

**DOI:** 10.3797/scipharm.1210-12

**Published:** 2012-11-22

**Authors:** Mashhour M. Ghanem, Saleh A. Abu-Lafi

**Affiliations:** 1 Pharmacare pharmaceutical Company, P.O. Box 677, Ramallah, Palestine.; 2 Faculty of Pharmacy, Al-Quds University, P.O. Box 20002, Abu-Dies, Palestine.

**Keywords:** Sulfadiazine sodium, Trimethoprim, Bactizine® forte injectable solution, Validation, Stability indicating method

## Abstract

A direct, precise, and stability-indicating HPLC method that is based on reversed-phase liquid chromatography (RP-HPLC) coupled with a photodiode array detector (PDA) was developed, optimized, and validated for the simultaneous determination of sulfadiazine sodium (SDZS) and Trimethoprim (TMP) in Bactizine® forte injectable solution. The separation was achieved using a C18 column (250 mm×4.6 mm i.d., 5 μm particle size) at room temperature, and an isocratic mobile phase that consisted of a trinary solvent mixture of water–acetonitrile–triethylamine (838:160:2, v/v) at pH 5.5 ± 0.05. The mobile phase was delivered at 1.4 ml/min and the analytes were monitored at 254 nm. The effects of the operational chromatographic conditions on the peak’s USP tailing factor, column efficiency, and resolution were systematically optimized. Forced degradation experiments were carried out by exposing SDZS, TMP standards, and their formulation to thermal, photolytic, oxidative, and acid-base hydrolytic stress conditions. The method was successfully validated in accordance to International Conference on Harmonization (ICH) and United States Pharmacopoeia (USP34/NF29) guidelines and found to be suitable for the quantitative determination and stability of SDZS and TMP in Bactizine® forte injectable solution.

## Introduction

**Fig. 1:**
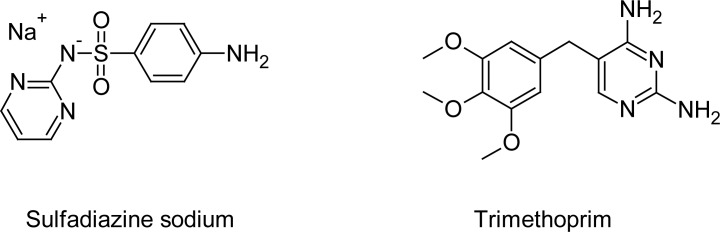
Chemical structures of sulfadiazine sodium (SDZS) and trimethoprim (TMP)

Bactizine® forte injectable solution is a synergistic combination of two antibacterial substances, sulfadiazine sodium (SDZS) and trimethoprim (TMP), at a concentration of 200mg of SDZS and 40mg of TMP per each milliliter of the injectable solution. It is recommended for the treatment of alimentary, respiratory, urinary tract, skin, and soft tissue infections caused by susceptible organisms, including E.coli, Enterobacter, Klebsiella, Streptococcus, Staphylococcus, Pasteurella, Clostridia, Salmonella, Shigella, Brucella spp., Actinomyces, Corynebacterium spp., Bordetella spp., Nisseria spp., Vibrio spp., and Proteus organisms. It is especially useful for the long-term and short-term treatment of chronic bacterial urinary tract infections. This drug combination can also be used for the eradication of coccidiosis in dogs and cats [1]. Figure 1 portrays the chemical structures of the two active ingredients, sulfadiazine sodium (SDZS) and trimethoprim (TMP) present in the Bactizine® forte injectable solution.

The determination of SDZS and TMP is official in both BP and USP as single components in which the HPLC method is described for each drug alone [2–5]. So far, no stability-indicating HPLC method has been reported for the direct simultaneous determination of SDZS and TMP in any formulation. However, there is a BP method that documented the determination of the combined active ingredients in the injectable form by using UV-Vis spectrometry exclusively [6]. The BP method describes the use of 0.1M sodium hydroxide solution to extract SDZS and the chloroform solvent to extract the TMP, followed by successive dilutions with acids or bases prior to UV-absorption measurements. The use of UV-Vis spectrophotometric measurements couldn’t be considered as a stability-indicating procedure since the degradation products will interfere with the absorption of the active ingredients. The extractions and the pretreatments adopted in the BP method reduce its accuracy and as a result, decrease recovery. Moreover, the method is time consuming, labor intensive, and utilizes expensive solvents that are hazardous to the environment.

In the literature, there are many HPLC methods that separate SDZS from TMP with other active combinations [7–11]. LC-MS and LC MS-MS technologies have been utilized lately for the multiresidual analysis of combined veterinary drugs containing SDZS and TMP. Samples from different origins such as wastewater [12], sewage sludge [13], baby food [14], chicken [15], eggs [16], and honey [17] were investigated. Although these papers described the separation of many active compounds including ours, their target was not the quality control application of any veterinary commercials containing these drugs, and neither their stress stability, which is exactly what this paper is focused on.

Therefore, there is a need to develop a direct, specific, and stability-indicating quality control method that allows for the simultaneous determination of SDZS and TMP in the Bactizine® forte injectable solution within a reasonable retention time as per ICH/USP validation norms [18, 19]. The method should provide information about the degradation products that could form during storage as a result of environmental factors such as light, humidity, and temperature. Besides, the forced degradation study is expected to help in establishing shelf lives, and facilitating the formulation of manufacturing and packaging to improve the injectable product.

## Results and discussion

### Method development and optimization

The aim of the developed RP-HPLC method was to resolve SDZS from TMP and from the placebo which consisted of water, benzyl alcohol, and glycerol to comply with the requirements of the system suitability test. The separation of all the components from the degradants formed during the stress study was a second aim. Different mobile phases have been employed in order to optimize the desired HPLC method. These mobile phases differ in the percentage of triethylamine (TEA) additive, pH, the organic solvents type, strength, and temperature. The best conditions selected were based on minimizing peak tailing, improving peak symmetry, column efficiency, resolution, and total analysis time.

To select an appropriate monitoring wavelength, individual standard solutions of 0.05 mg/ml for both SDZS and TMP were prepared and scanned by the UV-Vis spectrophotometer separately. The overlaid ultraviolet absorption spectra of the two active ingredients demonstrated that they shared an optimum response at a wavelength near 254 nm, and it was therefore chosen during the entire study.

Our first choice of the mobile phase was acetonitrile: water (30:70; v/v) at pH of 6.0. The SDZS peak shape was fairly accepted while the TMP peak was extremely broad with a tailing factor of more than 2.5 and a poor number of theoretical plates. Trying different percentages of acetonitrile and substituting it with methanol did not solve the band broadening and the low column efficiency of the TMP peak. Therefore, TEA modifier was added at different concentrations of 0.1, 0.15, 0.2, and 0.25% to improve TMP peak shape by reducing the hydrogen bonding between the TMP and the free silanols groups of the stationary phase. The best peak shape, tailing factor, and column efficiency was generated at 0.2% and 0.25%. 0.2% was selected throughout the whole study.

The effect of acetonitrile (ACN) strength at fixed TEA and pH on retention and resolution was investigated. It was found that increasing the ratio of ACN in the mobile phase dramatically decreased the retention and resolution. The effect of ACN strength was greatly pronounced on TMP in comparison to SDZS. A reversal of elution order was noticed upon increasing the ACN up to 35%. The optimal separation was achieved at a 16% threshold. At this level, a resolution of more than six with tailing factors of less than 1.3 for both active ingredients was produced.

The influence of using different pH’s from 3.5 to 6.5 on resolution and tailing factor was examined. Maximum resolution for both active ingredients was accomplished at pH 5.5.

Different temperatures of 15°C up to 35°C with 5°C increments were also evaluated. It was found that the temperature had a negligible influence on resolution and tailing factors, therefore room temperature was chosen. A typical HPLC chromatogram of the placebo, which consisted of water, benzyl alcohol, and glycerol and a freshly prepared mixture of SDZS, TMP, and benzyl alcohol preservative under the optimized conditions, is shown in Figures 2a,b.

### Method Validation

The optimized chromatographic conditions were validated according to the ICH/USP guidelines [18–19]. Parameters such as system suitability, specificity, linearity, range, accuracy (recovery), precision (repeatability and intermediate precision), and robustness were all validated.

### System suitability

The system suitability was determined by making six replicate injections of the standard solution and analyzing each active ingredient for its peak area, resolution, peak USP tailing factor, and number of theoretical plates. The proposed accepted criteria are NMT 2% for RSD%, NLT 2 for resolution, NMT 2 for USP tailing factor, and NLT 2000 for the number of theoretical plates. The system suitability results for a combined solution of 160 μg/ml SDZS and 32 μg/ml TMP showed an RSD % of less than 1.0% for both peak areas. The peak tailing factors of SDZS and TMP were 1.22 and 1.19 respectively. The number of theoretical plates for the peaks of SDZS and TMP were 3163 and 30785 respectively. A resolution factor (R_S_) of more than six was always achieved between SDZS and TMP peaks. This method met the accepted requirements.

### Specificity (placebo and forced degradation interference)

First the chromatograms of the placebo, standards, and sample test solutions were recorded at the same wavelength of 254 nm in order to check the specificity of the optimized method. The retention times of the SDZS and TMP sample solution peaks exactly match the peaks of the standard solutions. No peaks were found at these retention times in the placebo chromatogram (Fig. 2-a). Therefore, this method is suitable for the identification and quantification of the active ingredients in the Bactizine® forte injectable solution.

Afterwards, the specificity of the developed method was assessed by performing forced degradation studies on pure standards of active ingredients separately to indicate the initial results, and on samples of Bactizine® forte injectable solution in the presence of their potential degradants. The stress conditions employed for the degradation studies include UV light (254nm), heat (70°C), acid hydrolysis (1.0 N HCl), base hydrolysis (1.0 N NaOH), and oxidation (10% H_2_O_2_).

The sample stress solutions were analyzed against freshly prepared standards. The assay and purity check (at 10% height) for the stressed placebo, standards, and sample solutions were calculated (Table 1). The accepted criteria of the developed method was set to be considered specific and stability-indicating if there were no interference between the main peaks and any other peaks in the chromatogram. Moreover, the peak purity index for the main peak was set to have a minimum value of 0.99 and the UV spectrum of the main peaks of the tested sample should have been identical to that of the standards. Finally, no splitting would be accepted for the main peaks.

Except for the oxidative stress conditions which show extensive degradation, it was observed that SDZS and TMP standards had undergone a partial degradation under all other stress circumstances. There was no interference between the main active ingredients and any other peaks in the chromatogram and a resolution value of more than two was always achieved. The peak purity index for both active ingredients was found to be greater than 0.999, a higher value than the accepted limit. The UV spectrum of the main standard peaks is identical to the main peak of the freshly prepared pure standard solution in all cases studied.

Almost the same pattern of degradation was obtained for SDZS and TMP in their Bactizine® forte injectable solution samples. Figures (3a–e) show the chromatographic peak profiles of the active ingredients along with preservatives and the degradation products after exposing the Bactizine® forte injectable solution to the different stress conditions as in Table 1.

### Sensitivity

The sensitivity of the method was examined by establishing the limit of detection (LOD) and the limit of quantitation (LOQ) for SDZS and TMP at a signal-to-noise ratio of three and ten, respectively, by injecting a series of dilute solutions with known concentrations. The LOD values of 0.5μg/ml and 0.7μg/ml for SDZS and TMP were obtained, respectively, and the LOQ values were 1.6 μg/ml and 2.3 μg/ml for SDZS and TMP, respectively, with %RSD of less than four (accepted criteria in less than 10%).

### Linearity and range

Different amounts of SDZS and TMP in the range of 50% to 150% of the labeled amount (five concentration levels and three replicates each) were added to Bactizine® forte matrix (glycerol formal, benzyl alcohol, and water).

The linearity in the range of 80 to 240 μg/ml for SDZS and 16 to 48 μg/ml for TMP was investigated. The regression lines demonstrated linearity in the tested range. The regression analysis confirmed that the deviation of the y-intercept from zero was not significant; and the regression lines were linear with *R^2^* of 0.9994 and 0.9991 for SDZS and TMP respectively (Figures 4a,b).

### Accuracy (recovery)

Different concentrations of the two active ingredients were added to the placebo matrix and the accuracy was measured as reflected by recovery. The data obtained for the evaluation of linearity were used. The accuracy as reflected from recovery data and statistical evaluation for the assay of the two active ingredients is listed in Table 2. The average recovery data of SDZS and TMP showed results between 98.6% and 101.3% with RSD% of less than 1.20%, and therefore the accepted value of NMT 2% was fulfilled.

## Precision

### Repeatability

One laboratory analyst carried out the assay of SDZS and TMP on six determinations of a homogeneous sample of Bactizine® forte injection at the 100% level of the test concentration with the same analytical equipment on the same day. The repeatability results of the peak areas and statistical evaluation for the assay of the two active ingredients showed RSD% values of 0.78% and 0.91% for SDZS and TMP respectively.

### Intermediate Precision (ruggedness)

Two laboratory analysts carried out the assay of SDZS and TMP on twelve homogeneous samples of Bactizine® forte injectable solution at the 100% level of the final test concentration with different analytical equipments in two days. The assay results and statistical evaluation for the assay of the two active ingredients revealed RSD% values of 1.12% and 1.37% for SDZS and TMP respectively. The results of the assay of the two ingredients were within a suitable intermediate precision for the specified range.

### Robustness

Robustness of the proposed new method included six deliberate variations to some chromatographic parameters as summarized in Table 3. The modifications include different mobile phase flow rates of 1.2, 1.4, and 1.6 ml/min and three different column temperatures in the range 15–35°C. Different TEA percentages in the mobile phase (in the range of ± 5 of the nominal value and the normal % TEA) and different ACN percentages in the mobile phase (in the range of ± 5 of the nominal value and the normal % ACN) were also investigated. Three column batches filled with the same prescribed stationary phases were studied. Finally, three different pH values of the mobile phase at 5.3, 5.5, and 5.7 were tested. The RSD% values showed no significant change in the final assay results of each of the above two ingredients using the six variations (Table 3).

## Experimental

### Materials

Sulfadiazine sodium (SDZS) and trimethoprim (TMP) reference standards were purchased from Sigma-Aldrich (Germany). Glacial acetic acid, triethylamine (TEA), HPLC grade acetonitrile (ACN) and methanol (MeOH) solvents, hydrochloric acid fuming 37%, sodium hydroxide pellets, and hydrogen peroxide 30%, were purchased from Merck (Germany). Highly purified water was prepared by using a Millipore Milli-Q Plus water purification system. Bactizine® forte injectable samples, and all the active ingredients and excipients usually used in manufacturing the pharmaceutical combination, were kindly supplied by Pharmacare pharmaceutical company, Palestine.

### HPLC system

The LaChrom (Merck-Hitachi) high-performance liquid chromatograph equipped with an L-7100 pump, L-7200 autosampler, L-7300 column oven, DAD L-7450 photodiode array (PDA) detector, and D-7000 software HSM version 3.1 (Merck Hitachi, England) were employed. A double beam ultraviolet-visible spectrometer (PG Instruments, United Kingdom) was used.

### Chromatographic conditions

The chromatographic column used was the octadecyl silane C18 chemically bonded column (250 mm × 4.6 mm i.d., 5 μm particle) and purchased from ACE, United Kingdom. The optimum mobile phase was prepared by mixing the highly purified water with ACN and TEA (838:160:2; v/v), then allowed to equilibrate to room temperature, and then adjusted to a pH of 5.5 ± 0.05 with 0.2 N glacial acetic acid. The mobile phase was filtered using a 0.45 μm microporous filter and was degassed by sonication prior to use. A wavelength of 254 nm was chosen since it was found the most appropriate for the determination of the two active ingredients. The flow rate used was 1.4 ml/minute. The injection volume was 20 μl and the temperature of the column was room temperature. The total run time was only about 11 minutes.

### Preparation of standard solutions

The standard solution of SDZS and TMP was prepared by dissolving accurately weighed 200 mg of SDZS and 40 mg of TMP reference standards in 80 ml of MeOH, then shaken by mechanical means for five minutes, sonicated for two minutes, and then diluted up to 100 ml with the same solvent. Finally, 2 ml of this solution was pipetted into a 25 ml volumetric flask and completed to volume using the mobile phase. This solution was filtered using a 0.45 μm membrane filter. The obtained final solution contained 160 μg/ml SDZS and 32 μg/ml TMP. The solution was protected from light.

### Preparation of sample solution formulation

The sample solution was prepared by transferring 1 ml of Bactizine® forte injection to a 100 ml volumetric flask containing 80 ml of MeOH, shaken by mechanical means for five minutes, sonicated for two minutes, and then diluted up to 100 ml with the same solvent. Finally, 2 ml of this solution was pipetted into a 25 ml volumetric flask and completed to volume using the mobile phase. This solution was filtered using a 0.45 μm membrane filter. The obtained final solution contained 160 μg/ml SDZS and 32 μg/ml TMP. The solution was protected from light.

### Forced degradation study

#### Standard drug stock solutions

The forced degradation study was conducted on solutions that were prepared by transferring 200mg SDZS reference standard into five different 100 ml volumetric flasks. Also, 40 mg TMP reference standards were transferred separately into another five different 100ml volumetric flasks. Then 50 ml of MeOH was added in each flask and shaken by mechanical means for five minutes, and sonicated for two minutes until completely dissolved. These stock solutions were kept at room temperature, protected from light, and used for forced degradation studies.

#### Acid hydrolysis

Ten ml of 1.0 N HCl was added into one of the flasks containing the SDZS stock solution and another 10 ml was added into one of the flasks containing the TMP stock solution and kept at room temperature for 60 minutes in a dark place and then neutralized with 1.0 N NaOH and finally diluted to 100 ml with MeOH. Then 2 ml of this solution was pipetted into a 25 ml volumetric flask and completed to volume using the mobile phase. This solution was filtered using a 0.45 μm membrane filter. The obtained final solution contained 160 μg/ml SDZS and 32 μg/ml TMP.

#### Base hydrolysis

Ten ml of 1.0 N NaOH was added into one of the flasks containing the SDZS stock solution and another 10 ml was added into one of the flasks containing the TMP stock solution and kept at room temperature for 60 minutes in a dark place and then neutralized with 1.0 N HCl and finally diluted to 100 ml with MeOH. Then 2 ml of this solution was pipetted into a 25 ml volumetric flask and completed to volume using the mobile phase. This solution was filtered using a 0.45 μm membrane filter. The obtained final solution contained 160μg/ml SDZS and 32μg/ml TMP.

#### Oxidative hydrolysis

Ten ml of 10% H2O2 was added into one of the flasks containing the SDZS stock solution and another 10 ml was added into one of the flasks containing the TMP stock solution and kept at room temperature for 24 hours in a dark place and then diluted to 100 ml with MeOH. Then 2 ml of this solution was pipetted into a 25 ml volumetric flask and completed to volume using the mobile phase. This solution was filtered using a 0.45 μm membrane filter. The obtained final solution contained 160 μg/ml SDZS and 32 μg/ml TMP.

#### Thermal degradation

One of the flasks containing the SDZS stock solution and another one containing the TMP stock solution were studied separately for their thermal degradation by keeping them at 70°C in a water bath protected from light for 72 hours and then diluted to 100 ml with MeOH. Then 2 ml of this solution was pipetted into a 25 ml volumetric flask and completed to volume using the mobile phase. This solution was filtered using a 0.45 μm membrane filter. The obtained final solution contained 160 μg/ml SDZS and 32 μg/ml TMP.

#### Photo degradation

One of the flasks containing the SDZS stock solution and another one containing the TMP stock solution were studied separately for their photodegradation by exposing them to UV light at 254 nm for 48 hours and then diluted to 100 ml with MeOH. Then 2 ml of this solution was pipetted into a 25 ml volumetric flask and completed to volume using the mobile phase. This solution was filtered using a 0.45 μm membrane filter. The obtained final solution contained 160 μg/ml SDZS and 32μg/ml TMP.

#### Forced degradation study on Bactizine® forte injectable solution

The sample stock solutions were prepared by separately transferring 1 ml of the Bactizine® forte injectable solution (containing 200 mg SDZS and 40 mg TMP) into a series of five different 100 ml volumetric flasks. The very same procedure adopted for the standard solutions was used in the Bactizine® solution. The obtained final solution also contained 160 μg/ml SDZS and 32 μg/ml TMP.

## Conclusion

The validated HPLC method developed for the quantitative quality control determination of SDZS and TMP in Bactizine® forte injectable solution was evaluated over linearity, range, precision, system suitability, accuracy, specificity, ruggedness, and robustness. All the validation results were within the allowed specifications of the ICH/USP guidelines. The developed method is proven to be rapid, accurate, and stability-indicating for the simultaneous determination of the combined SDZS and TMP in Bactizine® forte injectable formulation in the presence of excipients and the degradation products. There was always a complete separation of both ingredients from their degradation products and from the placebo. As a result, the proposed HPLC method could be adopted for the quantitative quality control and routine analysis of Bactizine® fort injectable solution.

## Figures and Tables

**Fig. 2a:**
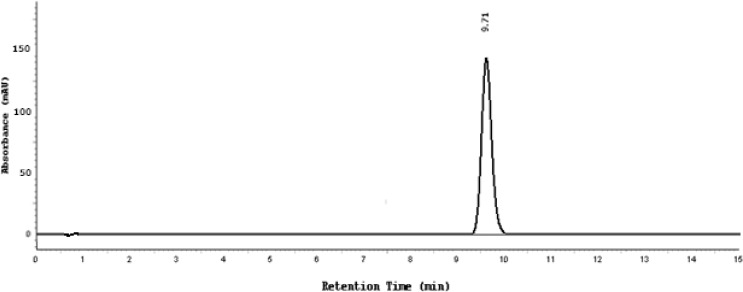
Typical chromatogram of the placebo, the peak at 9.7 minutes is due to benzyl alcohol

**Fig. 2b:**
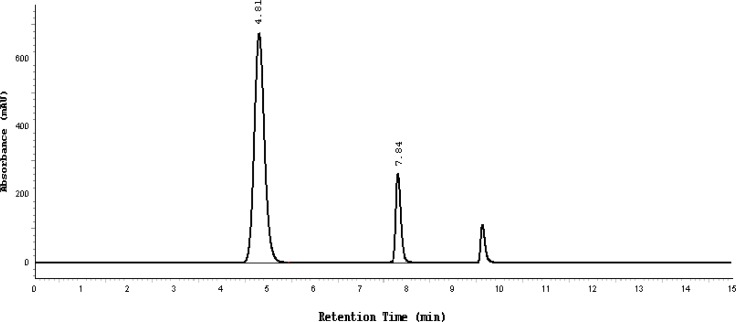
Typical chromatogram of a mixture of 160 μg/ml SDZS (4.81 minute) 32 μg/ml TMP (7.84 minutes), the last peak is due to benzyl alcohol

**Fig. 3a:**
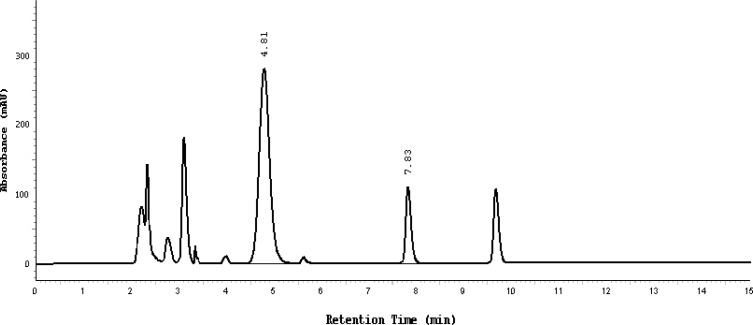
Chromatogram of oxidative degradation, SDZS (4.8 minute), TMP (7.8 minute), the last peak is due to benzyl alcohol, the other additional peaks are due to degradation products

**Fig. 3b:**
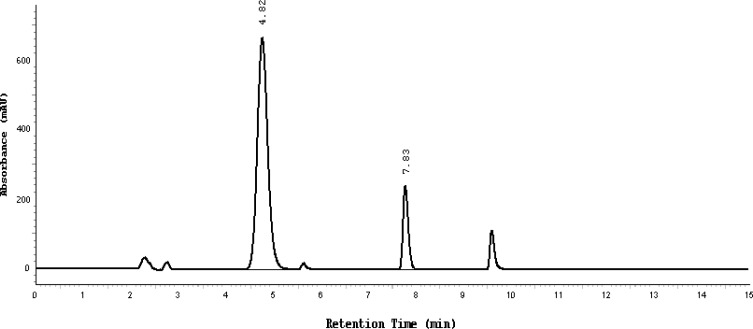
Chromatogram of UV degradation, SDZS (4.8 minute), TMP (7.8 minute), the last peak is due to benzyl alcohol, the other additional peaks are due to degradation products

**Fig. 3c:**
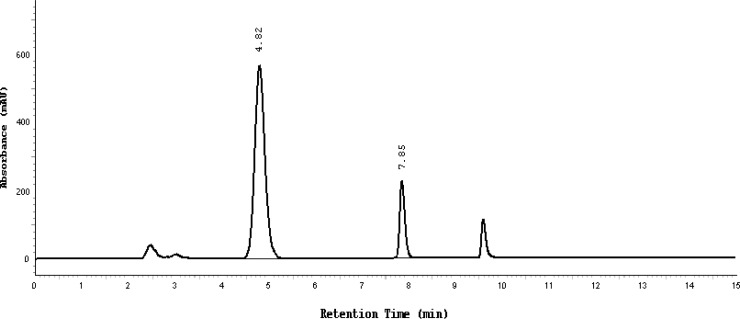
Chromatogram of acidic degradation, SDZS (4.8 minute), TMP (7.8 minute), the last peak is due to benzyl alcohol, the other additional peaks are due to degradation products

**Fig. 3d:**
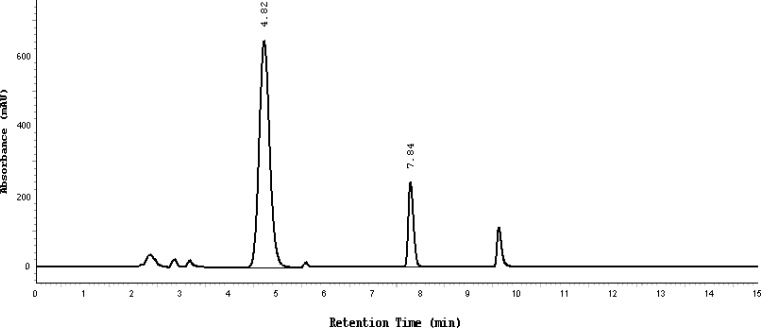
Chromatogram of thermal degradation, SDZS (4.8 min), TMP (7.8 min), the last peak is due to benzyl alcohol, the other additional peaks are due to degradation products

**Fig. 3e:**
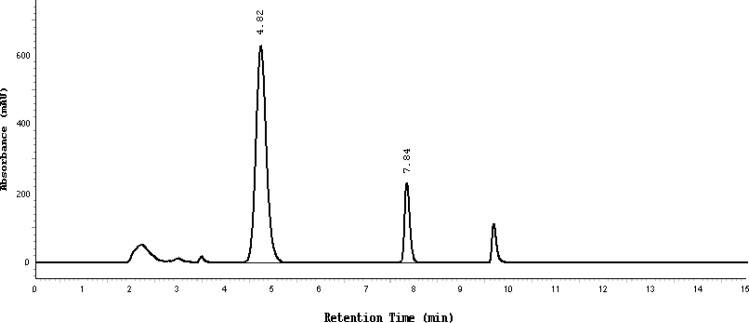
Chromatogram of basic degradation, SDZS (4.8 minute), TMP (7.8 minute), the last peak is due to benzyl alcohol, the other additional peaks are due to degradation products

**Fig. 4a:**
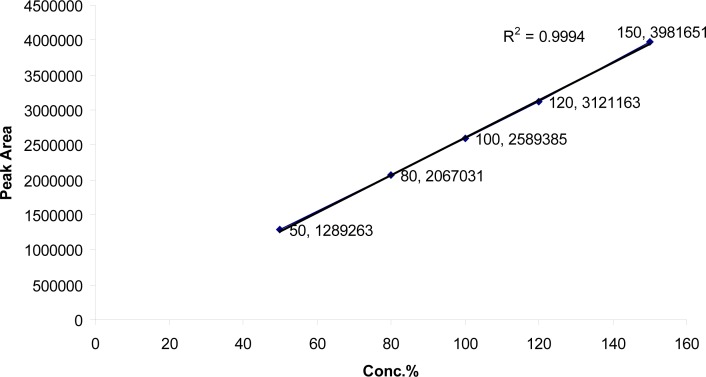
Linearity and range for Sulfadiazine Sodium

**Fig. 4b:**
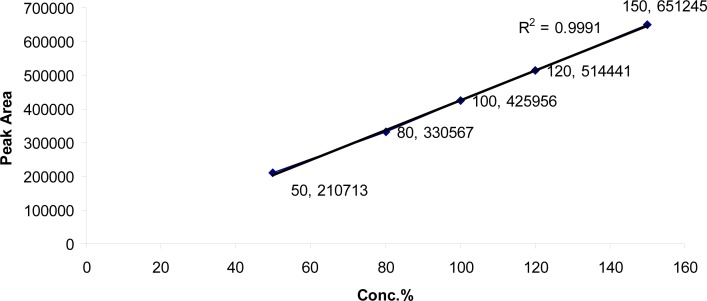
Linearity and range for Trimethoprim

**Tab. 1.** Summary of the forced degradation of SDZS and TMP standards and Bactizine® forte injectable solution

**Name**	**Stress condition**	**Degradation %**	**Peak purity index**
SDZS standard	Acidic/1.0 N HCl / 60 min at room temperature	10.47	0.9992
	Alkaline/1.0 N NaOH / 60min at room temperature	5.03	0.9996
	Oxidative/10 % H_2_O_2_ /24 hours at room temperature	51.04	0.9987
	Thermal/70 °C/72 hours	7.66	0.9992
	Light/ UV-254nm /48 hours	3.76	0.9993

SDZS sample	Acidic/1.0 N HCl / 60 min at room temperature	10.83	0.9999
	Alkaline/1.0 N NaOH / 60min at room temperature	5.42	0.9999
	Oxidative/10 % H_2_O_2_ /24 hours at room temperature	51.77	0.9995
	Thermal/70 °C/72 hours	8.04	0.9997
	Light/ UV-254nm /48 hours	3.91	0.9999

TMP standard	Acidic/1.0 N HCl / 60 min at room temperature	16.2	1.0000
	Alkaline/1.0 N NaOH / 60min at room temperature	10.16	1.0000
	Oxidative/10 % H_2_O_2_ /24 hours at room temperature	26.1	0.9999
	Thermal/70 °C/72 hours	12.93	1.0000
	Light/ UV-254nm /48 hours	13.2	0.9999

TMP sample	Acidic/1.0 N HCl / 60 min at room temperature	15.7	1.0000
	Alkaline/1.0 N NaOH / 60min at room temperature	10.85	0.9998
	Oxidative/10 % H_2_O_2_ /24 hours at room temperature	25.6	0.9999
	Thermal/60 °C/72hours	12.34	0.9998
	Light/ UV-254nm /48 hours	14.2	1.0000

**Tab. 2.** Average recoveries, RSD% values at five concentration levels of spiking (*n=3*) of SDZS and TMP

**Active ingredient**	**Amount added (level %)**	**Average recovery (%) ± S.D. *(n=3)***	**RSD (%)**
SDZS	80 μg /ml (50%)	98.9 ± 0.62	0.63
	128 μg /ml (80%)	99.4 ± 0.81	0.81
	160 μg /ml (100%)	99.7 ± 0.58	0.58
	192 μg /ml (120%)	100.4 ± 1.13	1.13
	240 μg /ml (150%)	101.3 ± 1.05	1.04

TMP	16 μg/ml (50%)	98.6 ± 1.16	1.18
	25.6 μg/ml (80%)	98.9 ± 0.96	0.97
	32 μg/ml (100%)	99.8 ± 0.84	0.84
	38.4 μg/ml (120%)	99.6 ± 1.08	1.08
	48 μg/ml (150%)	100.8 ± 0.99	0.98

**Tab. 3.** Robustness testing of the two active ingredients of SDZS and TMP

**Active ingredient**	**Parameter**	**Average assay % ± S.D. *(n=3)***	**RSD % *(n=3)***
SDZS	Flow rate (ml/min)	98.7 ±1.00	1.01
	Temperature (°C)	99.4 ±0.86	0.87
	% TEA buffer	98.5 ±1.45	1.47
	% ACN	101.4 ±1.43	1.41
	Column batches	100.8 ±0.93	0.92
	Mobile phase pH	99.8 ±0.91	0.91

TMP	Flow rate (ml/min)	100.4 ±0.97	0.97
	Temperature (°C)	99.2 ±0.88	0.89
	% TEA buffer	98.6 ±1.39	1.41
	% ACN	100.8 ±1.57	1.56
	Column batches	101.6 ±1.18	1.16
	Mobile phase pH	99.2 ±0.83	0.84
